# Supplementary material for: Ultra-Thin SnS2-Pt Nanocatalyst for Efficient Hydrogen Evolution Reaction
Source: Nanomaterials (Basel). 2020 Nov 25;10(12):2337. doi: 10.3390/nano10122337 (PMC7760803; doi:10.3390/nano10122337)
Supplement: Supplementary file 1 [file nanomaterials-10-02337-s001.pdf]

Supplementary Materials

Ultra-thin SnS_2 Nanocatalyst for Efficient Hydrogen Evolution Reaction

Yanying Yu [1], Jie Xu [1], Jianwei Zhang, Fan Li, Jiantao Fu, Chao Li *, Cuihua An *

Center for Electron Microscopy, TUT-FEI Joint Laboratory, Tianjin Key Laboratory of Advanced Porous Functional Materials, Institute for New Energy Materials & Low-Carbon Technologies, School of Materials Science and Engineering, Tianjin University of Technology, Tianjin 300384, China.

EXPERIMENTAL SECTION

Pt-SnS_2 nanosheets synthesis. 0.35 g of crystalline tin tetrachloride and 0.3 g of thioacetamide (molar ratio 1:4) were dissolved in 40 ml of isopropyl alcohol and stirred continuously for 30 minutes to form a clear solution which was transferred to a 40 ml teflon-lined stainless steel autocloner, then sealed and heated at 180°C for 24 h. Then, the substrate covered with the precursor was rinsed several times with deionized water and ethanol, centrifuged and dried at 60°C for 12 h to obtain ultra-thin nanometer SnS_2.

The SnS_2 powder prepared above and hexahydrate of chloroplatinic acid (1 mmol/L) were dissolved in anhydrous ethanol at a mass ratio of 50:1, stirred continuously for 3 hours, centrifuged and rinsed with deionized water for several times, dried at 12 h at 60°C and calcined in vacuum at Ar 200°C for 2 h to obtain SnS_2-Pt.

Supplementary Figures

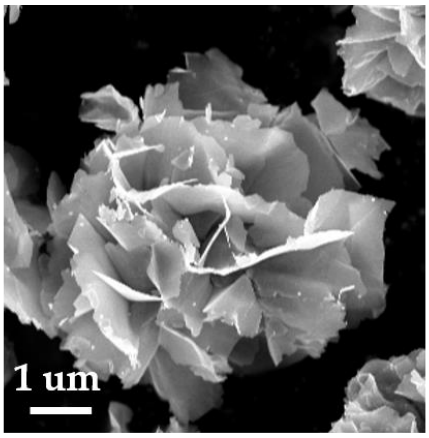

(a)

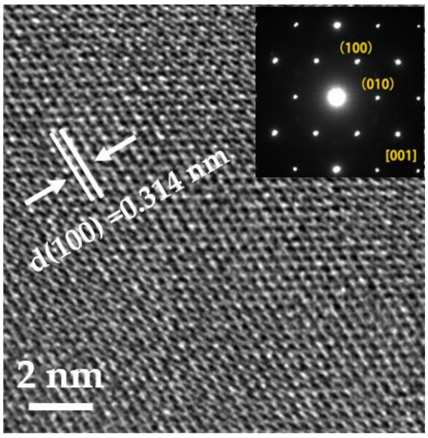

(b)

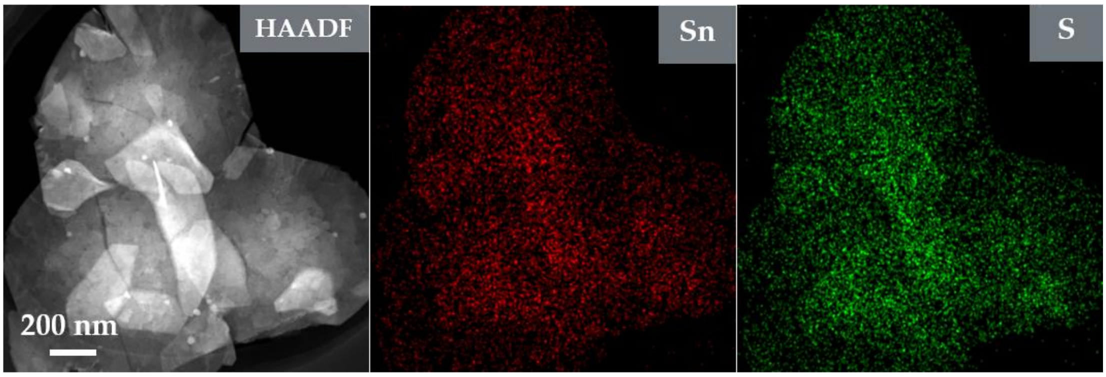

(c)

Figure S1. Nanosheet structure of pure SnS_2. (a) SEM images. (b) TEM images of low-power morphologies. (c) High-resolution transmission diagram, where is the corresponding SAED diagram. (d) HAADF image and EDX mapping of Sn and S in pure SnS_2 nanometer tablets.

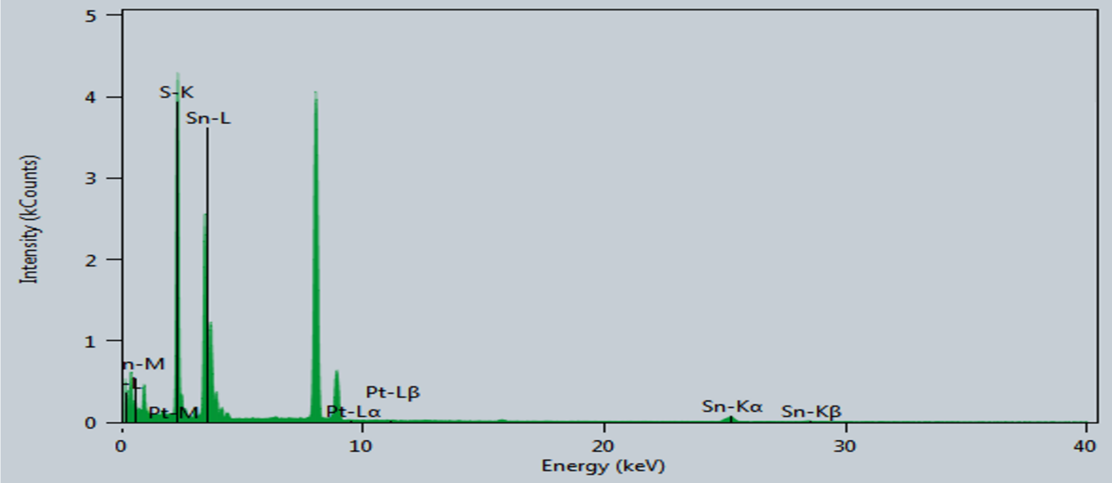

Z	Element	Family	Atomic Fraction(%)	Atomic Error(%)	Mass Fraction(%)	Mass Error(%)	Fit error(%)
16	S	K	66.49	7.25	34.72	2.53	0.26
50	Sn	L	33.12	5.16	64.03	8.51	0.28
78	Pt	L	0.39	0.06	1.25	0.17	2.74

Table S1. SnS_2-Pt-3 nanosheet EDS and corresponding element ratio.

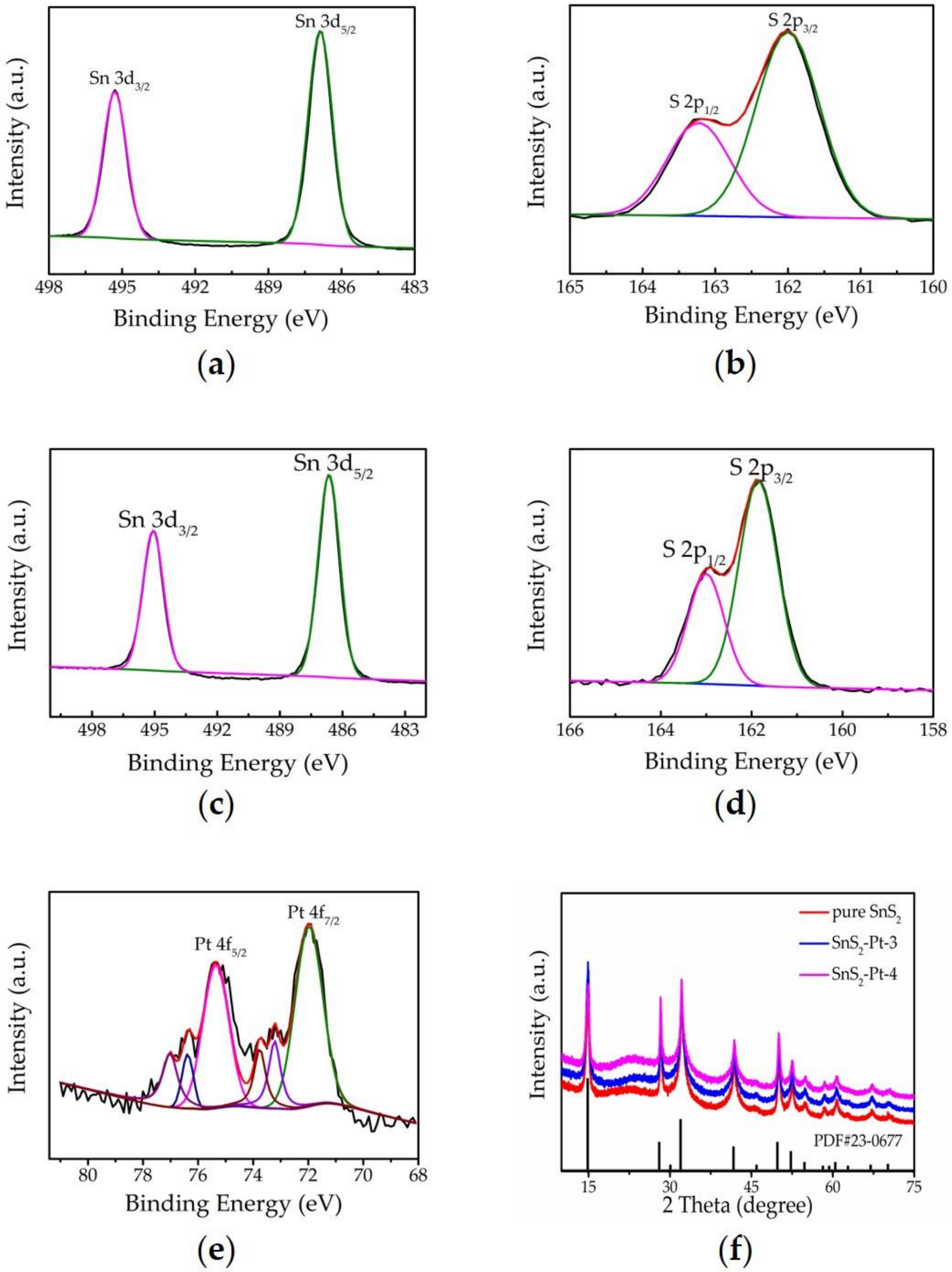

Figure S2. Chemical structure analysis of pure SnS_2 and SnS_2-Pt-4. (a, b) High-resolution XPS spectra of pure SnS_2. (c, d and e) High-resolution XPS spectra of SnS_2-Pt-4. (f) XRD of pure SnS_2, SnS_2-Pt-3 and SnS_2-Pt-4.

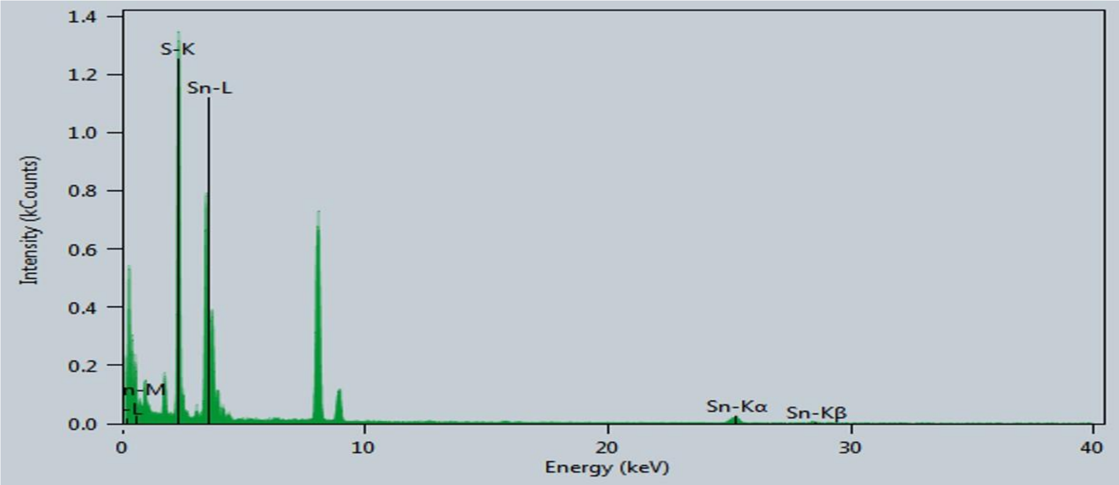

Z	Element	Family	Atomic Fraction(%)	Atomic Error(%)	Mass Fraction(%)	Mass Error(%)	Fit error(%)
16	S	K	67.40	7.38	35.84	2.63	0.82
50	Sn	L	32.60	5.08	64.19	8.53	0.16

Table S2. Pure SnS_2 nanosheet EDS and corresponding element ratio.

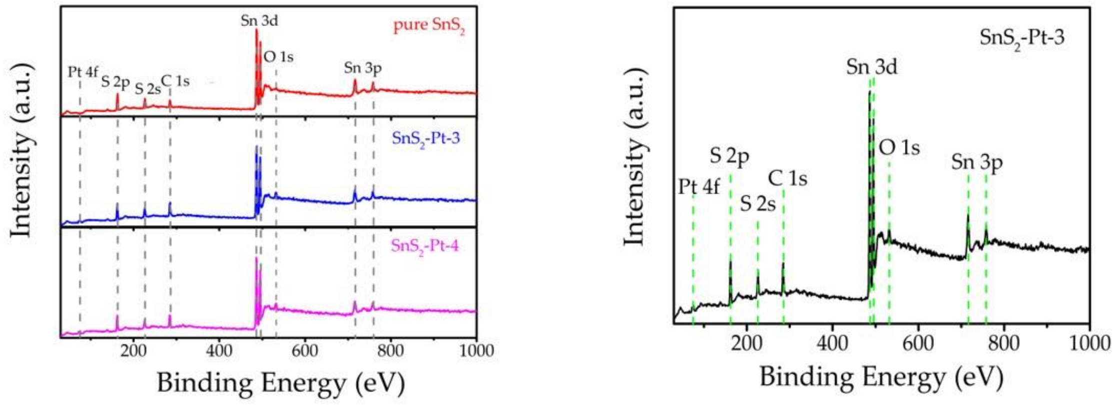

(a) (b)

Figure S3. The whole XPS spectrum. (a) Pure SnS_2, SnS_2-Pt-3 and SnS_2-Pt-4. (b) SnS_2-Pt-3 after 20-hour test.

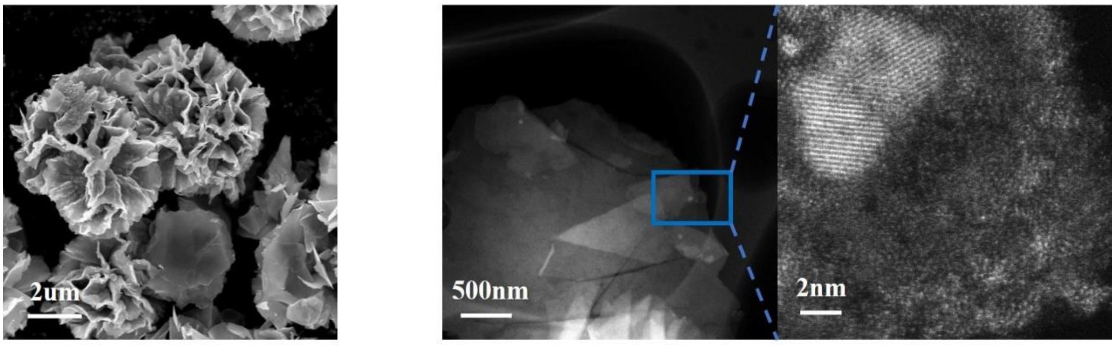

(**a**) (**b**)

Figure S4. (a) SEM and (b) TEM of SnS_2-Pt-4.

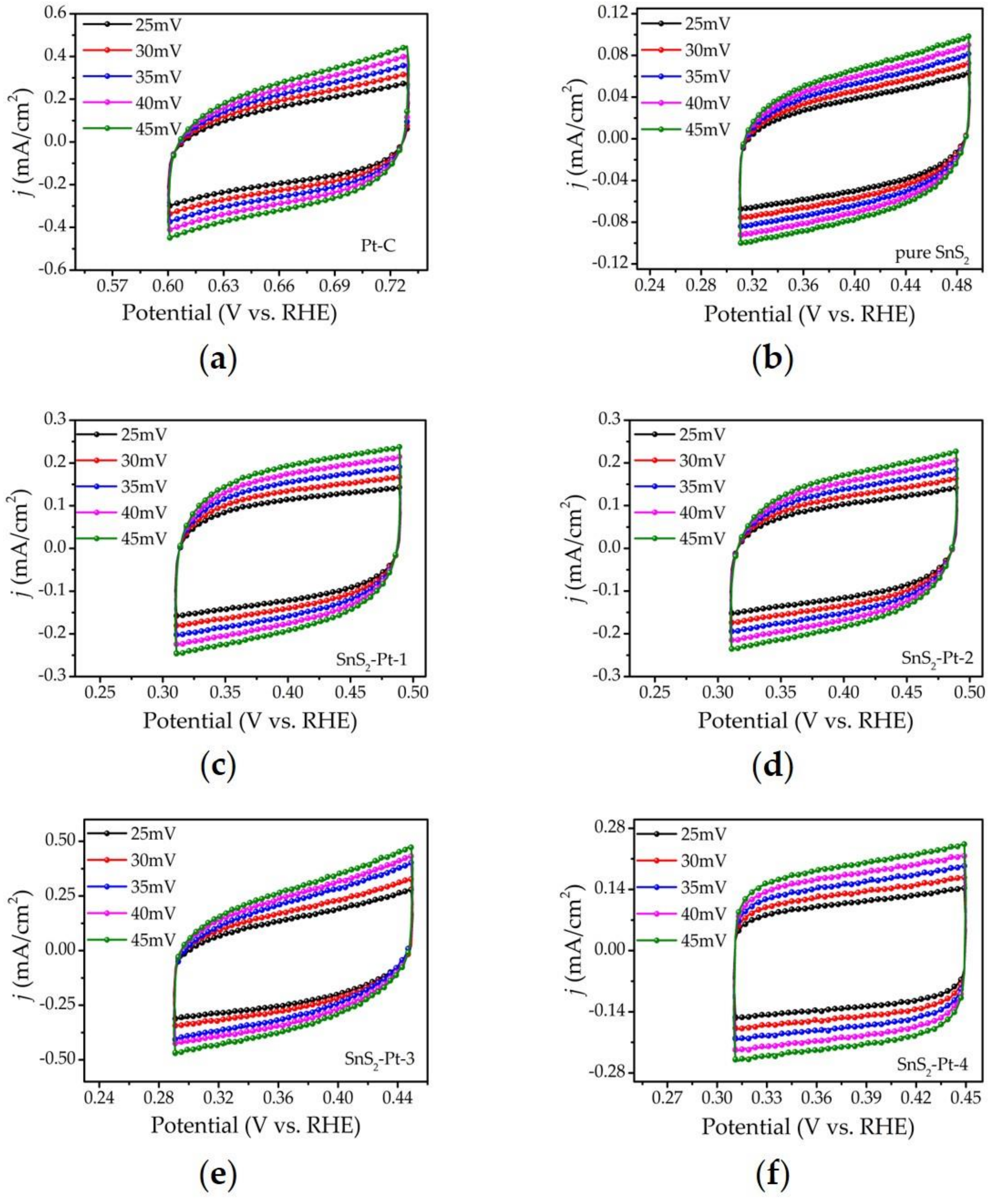

Figure S5. Double-layer capacitance measurements. Select the non-Faraday region and measure the CV curves of Pt-C, pure SnS$_2$, SnS$_2$-Pt-1, SnS$_2$-Pt-2, SnS$_2$-Pt-3 and SnS$_2$-Pt-4 at scan rates of 25, 30, 35, 40, and 45 mV s^{-1}.

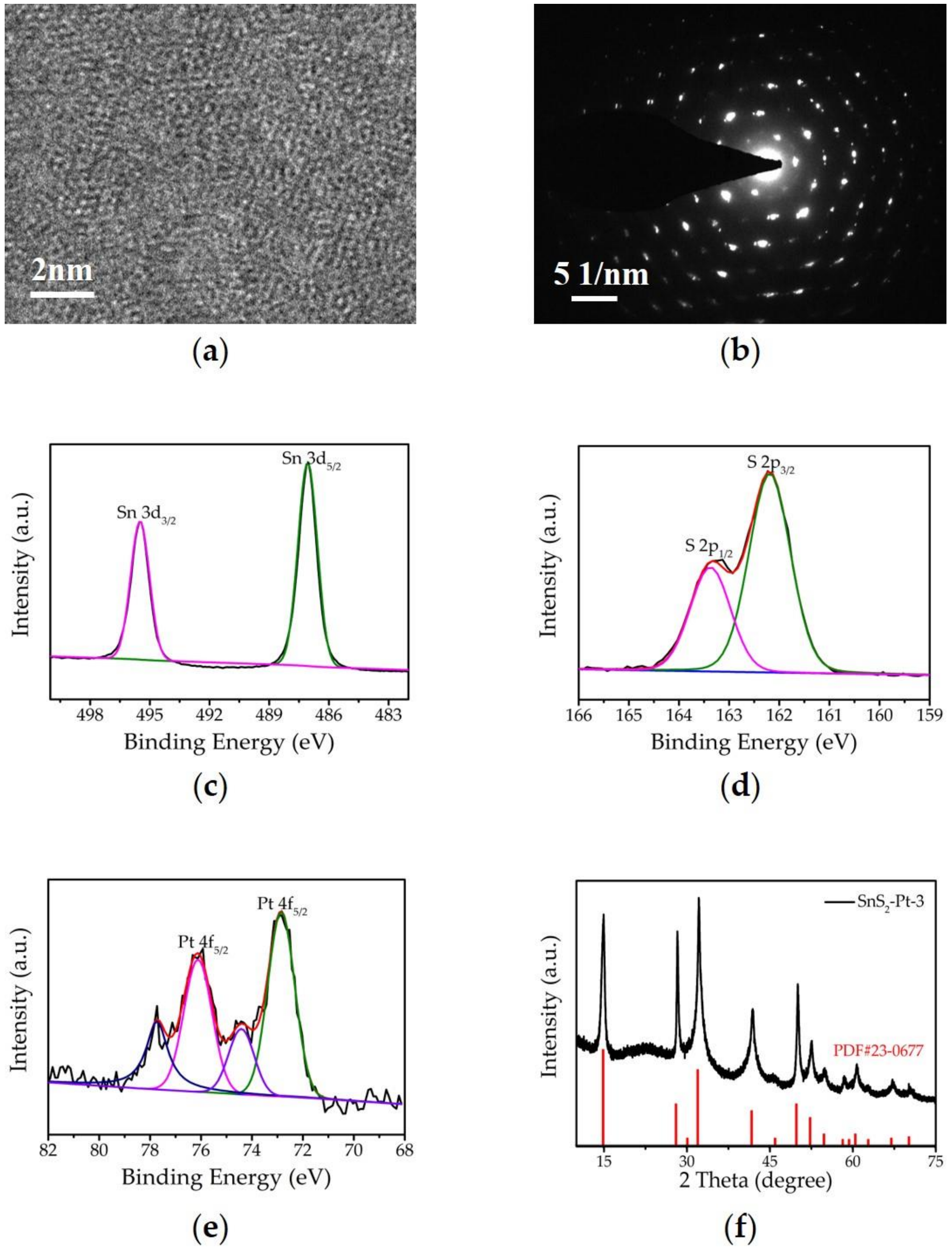

Figure S6. Structure and performance characterization diagram of SnS_2-Pt-3 after 20-hour test. (a, b) High-resolution transmission diagram, in which is the corresponding SAED pattern. (c, d and e) High-resolution XPS spectra. (f) XRD.
